# *Bifidobacterium bifidum* CCFM1163 Alleviated Cathartic Colon by Regulating the Intestinal Barrier and Restoring Enteric Nerves

**DOI:** 10.3390/nu15051146

**Published:** 2023-02-24

**Authors:** Nan Tang, Qiangqing Yu, Chunxia Mei, Jialiang Wang, Linlin Wang, Gang Wang, Jianxin Zhao, Wei Chen

**Affiliations:** 1 State Key Laboratory of Food Science and Technology, Jiangnan University, Wuxi 214122, China; 2 School of Food Science and Technology, Jiangnan University, Wuxi 214122, China; 3 (Yangzhou) Institute of Food Biotechnology, Jiangnan University, Yangzhou 225004, China; 4 National Engineering Research Center for Functional Food, Jiangnan University, Wuxi 214122, China

**Keywords:** *Bifidobacterium bifidum*, cathartic colon, intestinal barrier, enteric nervous system

## Abstract

Cathartic colon (CC), a type of slow-transit constipation caused by the long-term use of stimulant laxatives, does not have a precise and effective treatment. This study aimed to evaluate the ability of *Bifidobacterium bifidum* CCFM1163 to relieve CC and to investigate its underlying mechanism. Male C57BL/6J mice were treated with senna extract for 8 weeks, followed by a 2-week treatment with *B. bifidum* CCFM1163. The results revealed that *B. bifidum* CCFM1163 effectively alleviated CC symptoms. The possible mechanism of *B. bifidum* CCFM1163 in relieving CC was analyzed by measuring the intestinal barrier and enteric nervous system (ENS)-related indices and establishing a correlation between each index and gut microbiota. The results indicated that *B. bifidum* CCFM1163 changed the gut microbiota by significantly increasing the relative abundance of *Bifidobacterium, Faecalibaculum, Romboutsia,* and *Turicibacter* as well as the content of short-chain fatty acids, especially propionic acid, in the feces. This increased the expression of tight junction proteins and aquaporin 8, decreased intestinal transit time, increased fecal water content, and relieved CC. In addition, *B. bifidum* CCFM1163 also increased the relative abundance of *Faecalibaculum* in feces and the expression of enteric nerve marker proteins to repair the ENS, promote intestinal motility, and relieve constipation.

## 1. Introduction

Cathartic colon (CC), a type of slow-transit constipation, is generally caused by the long-term use of stimulant laxatives such as anthraquinones [1]. However, the clinical treatment for CC remains the same as that for common constipation, with no specifically effective treatment. In severe cases, relief can only be provided through surgery.

Previously, we successfully constructed a CC constipation model using senna extract and found that CC mice have symptoms of slow-transit constipation, along with damage to the intestinal mechanical barrier and enteric nervous system (ENS) [2]. *Bifidobacterium bifidum* CCFM1163 was found to alleviate CC after the application of different probiotic interventions. Thus, this study aimed to determine the potential mechanism of *B. bifidum* CCFM1163 in alleviating damage to the intestinal barrier and ENS and provide a theoretical basis for the development and application of bifidobacterial products that can prevent and treat constipation.

An intact intestinal barrier effectively defends against the invasion of foreign pathogenic bacteria in the intestinal lumen and plays a key role in maintaining intestinal homeostasis and health. The intestinal barrier is divided into biological, chemical, mechanical, and immune barriers from the lumen to the outside of the intestine [3]. Several animal and clinical studies have demonstrated that probiotics can positively affect the intestinal barrier. For example, *Bifidobacteria* increase the relative abundance of *Lactobacillus* and decrease the relative abundance of pathogenic bacteria (*Alistipes, Odoribacter,* and *Clostridium*) in the host intestine, thereby affecting the biological barrier and relieving constipation [4]. Another clinical study found that *B. bifidum* CCFM16 modulates the host biological barrier and effectively relieves chronic constipation in adults [5]. Moreover, *B. bifidum* can promote intestinal motility in constipated mice by influencing gastrointestinal active peptide levels and 5-hydroxytryptamine (5-HT) receptor expression in the chemical barrier [6]. Several studies have demonstrated that probiotics alleviate disease by affecting the intestinal mechanical barrier. For example, probiotics enhance the intestinal mechanical barrier by directly upregulating the gene expression of tight junction (TJ) proteins and MUC2 (mucin2) [7,8] and competitively excluding the binding of intestinal pathogens to the mucosa [9,10]. The fermentation supernatant of probiotic bacteria has the same effect. For instance, *Lactobacillus rhamnosus* fermentation supernatant modulates 5-HT receptor 4 (5-HT_4_R) and the gut microbiota, which in turn promotes the production of intestinal mucin [11]. A recent study demonstrated that *B. longum* reduces inflammation and relieves constipation by downregulating interleukin-1β (IL-1β) and tumor necrosis factor-α (TNF-α) expression in colonic tissues [12].

The ENS comprises enteric neurons and enteric glial cells (EGCs). Protein gene product 9.5 (PGP9.5) is a specific marker of enteric neurons. Moreover, EGCs secrete self-marker proteins, such as glial fibrillary acidic protein (GFAP) as well as S100β, and Sox10 proteins, which are frequently used to identify EGCs. Studies have revealed that ENS injury causes intestinal motility disorders and that the beneficial effects of probiotics on intestinal motility are partly mediated by ENS [13,14,15]. On the one hand, probiotics can increase the expression of GFAP, S100β, and Sox10 in the intestinal mucosa, as well as that of neurotransmitters in the submucosal nerve plexus [16,17]. On the other, they can modulate enteric neurons and certain neuronal subtypes and promote intestinal motility by upregulating Toll-like receptor 2 (TLR2) expression in enteric neurons [18,19].

Based on these studies, this study aimed to determine the effect of *B. bifidum* CCFM1163 on CC and its possible mechanisms through the biological, chemical, mechanical, and immune barriers and the ENS. This study provides a new theoretical basis for the creation of functional probiotics with independent intellectual property rights, and new ideas for the development of functional foods to improve national health.

## 2. Materials and Methods

### 2.1. Bacterial Treatment

The three strains of *B. bifidum* (*B. bifidum* 45M3 [CCFM1163], *B. bifidum* M3 and *B. bifidum* M7) used in this experiment were isolated from healthy human feces and stored in the food microbial strain bank of Jiangnan University. The strains were retrieved from storage tubes at −80 °C and cultured after two generations of activation in modified de Man, Rogosa, and Sharpe broth with 0.05% (*w*/*v*) L-cysteine at 37 °C under anaerobic culture conditions. The cultured suspension was collected after centrifugation (8000× *g*, 4 °C, 15 min) and resuspended after discarding the supernatant followed by washing twice with phosphate-buffered saline (PBS) buffer. A quantity of 1 mL was used to determine the bacterial concentration by gradient dilution method, and the remaining suspension was stored at −80 °C. Before oral administration, the cells were diluted with PBS to a concentration of 5 × 10^9^ CFU/mL.

### 2.2. Animal Experiments

**Figure 1:**
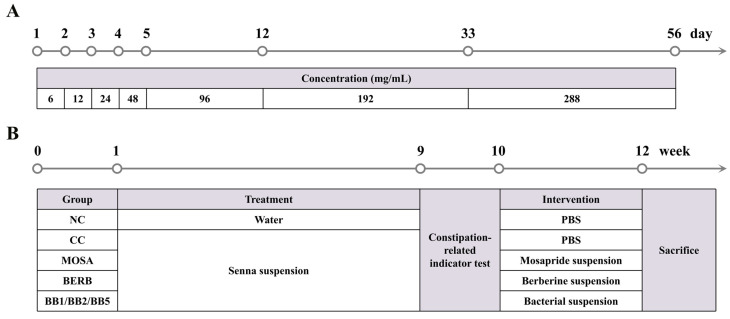
Schematic overview of the animal experiment. (**A**) Schematic of the senna suspension treatment procedure. (**B**) Schematic of the animal treatment procedure.

Eight-week-old male C57BL/6J mice were bought from the Model Animal Research Centre of Vital River (Shanghai, China). The animal experiments involved in this research were conducted at the Experimental Animal Center of Jiangnan University and approved by the Ethics Committee of Experimental Animals of Jiangnan University (JN.No 20210530c1201226[138]). All animals ate standard feed and drank water freely. After one week of adaptation, the mice were randomly divided into the following groups: normal control group (NC), cathartic colon group (CC), mosapride-treated group (MOSA), berberine-treated group (BERB), *B. bifidum* CCFM1163-treated group (BB1), *B. bifidum* M3-treated group (BB2), and *B. bifidum* M7-treated group (BB5) (n = 6/group). Senna extract (Xuhuang Biology Co., Ltd., Xi’an, China) was orally administered to all mice except NC mice, and the gavage volume of each mouse was 200 μL per day [20]. The specific method used is illustrated in Figure 1A. Subsequently, a few indices were measured in the following 1 week to determine the indications of constipation symptoms in the mice. After confirmation, the positive drug treatment groups were administered 200 μL of mosapride and berberine solutions (0.2 and 10 mg/mL, respectively) per day; bacterial treatment groups were administered 200 μL of different bacterial suspensions; and the NC group was administered 200 μL of sterile PBS by gavage once a day for 2 weeks. The timing of the animal treatment is shown in Figure 1B.

### 2.3. Determination of Constipation-Related Indicators

Gut transit time. The gut transit time refers to the total time it takes for the chyme to pass from the stomach through the intestine and then reach the anus and finally be excreted as feces. It reflects the peristaltic capacity of the entire gastrointestinal tract. The Evans blue test was used to evaluate gut transit [21]. To ensure the accuracy of the assay results, mice were fasted for 12 h and watered freely before the assay. In the beginning, mice were administrated 0.2 mL of Evans Blue semiliquid solution (2.5% Evans Blue and 1% methylcellulose) by gavage. The time interval between finishing the gavage and the expulsion of the first blue pellet was recorded as the gut transit time for each mouse.

Small intestine transit rate. Gum arabic was added to water as a thickener, heated, and boiled until the solution was clear. Activated carbon was then added to the boiling mixture to ensure uniform mixing. After cooling, the solution was diluted and fixed with water to 1000 mL to obtain an activated carbon solution. The mice were fasted for 12 h and watered freely before the assay. At the beginning, each mouse was administered 0.2 mL of activated carbon solution. Mice were free-ranged for 30 min and sacrificed, and their small intestinal segments were removed. The distance between the front section of the activated carbon and the total length of the small intestine were measured [22]. The small intestine transit rate was calculated by the following equation:

Small intestine transit rate (%) = Length of activated carbon propulsion (cm)/Total length of the small intestine (cm) × 100%(1)

Fecal water content. Feces were collected individually and weighed before and after freeze-drying [23]. The fecal water content was calculated according to the following equation:

Fecal water content (%) = (Wet weight of the feces (g) − Dry weight of the feces (g))/Wet weight of the feces (g) × 100%(2)

### 2.4. Histopathological Analysis

Terminal colon tissue (0.5 cm) was collected, immediately rinsed with pre-cooled saline, and then placed in 4% paraformaldehyde solution for fixation to avoid secondary damage to tissues. The fixed tissues were rinsed and dehydrated in 70%, 80%, and 90% ethanol solution (*v*/*v*) for 30 min and subsequently placed in a mixture of alcohol and xylene (alcohol: xylene = 1:1) and rinsed thrice. The sections were then transferred to a mixture of xylene and paraffin wax (xylene: paraffin wax = 1:1) for wax immersion. After paraffin embedding, 5 μm sections were created. The sections were placed on slides and stained with hematoxylin and eosin (H&E) following standard procedures. Finally, after sealing and complete solidification using neutral gum, the sections were photographed in a digital tissue section scanner (3DHistech, Budapest, Hungary). The histopathological scoring table of the colon is presented in the Supplementary Materials.

### 2.5. Immunofluorescence

The samples were embedded and sectioned using the previously described method. The sections were maintained at 60 °C for 60 min, immersed in xylene for 10 min, and then sequentially into 100%, 95%, 85%, and 75% ethanol (*v*/*v*) for 5 min. The mixture was then soaked for 5 min in deionized water thrice. The sections were added to 10 mmol/mL citrate buffer for 15 min to ensure the complete submersion of the tissue. Subsequently, the sections were soaked for 5 min in triethanolamine-buffered saline (TBS) and washed thrice. After air-drying, 50 μL of blocking buffer was added to block the sections for 30 min. In total, 50 μL of primary antibody (1:5000, ab7260, Abcam, Shanghai, China) was then added to each section and stained overnight at 4 °C. The next day, the same washing process was performed with TBS to remove liquid from the tissue. After drying, 20 μL of fluorescently labeled secondary antibody (1:500, ab150077, Abcam, Shanghai, China) was added and the sections were incubated for 60 min in the dark. After washing, DAPI was added to each section for 5 min, followed by washing with TBS. Finally, sealing buffer was added and coverslips were placed to seal the slides. Observation in a digital tissue section scanner (3DHistech, Budapest, Hungary).

### 2.6. Real-Time Polymerase Chain Reaction

Colon tissues were placed in enzyme-free centrifuge tubes containing enzyme-inactivating zirconia beads, and 1 mL TRIzol (Invitrogen, Carlsbad, CA, USA) was added for total RNA extraction. The extracted RNA was reverse transcribed into cDNA using a reverse transcription kit (Vazyme Biotech Co., Ltd., Nanjing, China). PCR systems were prepared according to the instructions of the qPCR mix (Bio-Rad, Hercules, CA, USA), and the PCR systems were used in a BioRad-CFX384 fluorescent quantitative gene amplification instrument (Bio-Rad, USA). Real-time qPCR was performed to detect the transcript levels of PGP9.5, S100β, GFAP, MUC2, zonula occluden-1 (ZO-1), Occludin, Claudin-1, Claudin-4, TNF-α, IL-1β, IL-6, tryptophan hydroxylase 1 (Tph1), 5-HT_2B_, 5-HT_4_, aquaporin 4 (AQP4), AQP8, G protein-coupled receptor 41 (GPR41), and GPR43 genes in the mouse colon. The primer sequences are provided in the Supplementary Materials.

### 2.7. Enzyme-Linked Immunosorbent Assay

The tissue was first rinsed with pre-cooled PBS to remove any residual blood and then placed in a centrifuge tube containing sterilized zirconia beads. PBS was added in a weight-to-volume ratio of 1:9 and tissue disrupted. The tissue was centrifuged at 4 °C at 5000× *g* for 10 min, and the supernatant was removed for the test. The concentrations of TNF-α, IL-1β, and IL-6 in colonic tissues were measured using a mouse ELISA kit (R&D, Minneapolis, MN, USA). A double antibody sandwich ELISA (Enzyme-linked Biotechnology Co., Ltd., Shanghai, China) was used to detect 5-HT in the tissues.

### 2.8. Short-Chain Fatty Acid (SCFA) Analysis

The contents of acetic acid (AA), propionic acid (PA), and butyric acid (BA) in feces were analyzed by gas chromatography–mass spectrometry (GC-MS). The stool samples were weighed and placed in 2 mL centrifuge tubes and homogenized with a tissue homogenizer after adding 500 μL of saturated sodium chloride and 40 μL of 10% sulfuric acid sequentially. Diethyl ether (1 mL) was added to the homogenized sample in a fume hood, mixed thoroughly, and centrifuged at 4 °C at 14,000× *g* for 15 min, and the supernatant was removed. The sample was then transferred to a centrifuge tube and allowed to stand for 15 min after adding 0.25 g anhydrous sodium sulfate to remove water from the sample. After centrifugation, 500 μL of the sample was analyzed using a gas chromatograph–mass spectrometer (GC-MS) (QP2010 Ultra; Shimadzu, Kyoto, Japan). The GC-MS analysis parameters were obtained from a previous study [24].

### 2.9. Gut Microbiota Analysis

Microbial genomic DNA was extracted from stool samples using a FastDNA^®^Spin Kit for Stool (MP Biomedicals, Santa Ana, CA, USA). The V3-V4 regions of the 16S rRNA gene were amplified using universal primers (341F and 806R). The PCR products were purified according to the instructions of the TIANgel Mini Purification Kit (Tiangen, Beijing, China), and DNA was quantified and mixed using the Qubit dsDNA Assay Kit (Life Technologies, Invitrogen, Carlsbad, CA, USA). The amplicons were sequenced on the MiSeq PE300 platform using a MiSeq kit (Illumina, San Diego, CA, USA).

Data processing and bioinformatics analysis were carried out using the QIIME2 platform. The β-diversity was visualized by principal coordinate analysis (PCoA) using an online website (https://www.microbiomeanalyst.ca/, accessed on 10 January 2023) [25]. Linear discriminant analysis effect size (LEfSe) was used to calculate the differential abundance of microbial taxa, and taxonomic cladogram trees were drawn using an online website (http://huttenhower.sph.harvard.edu/galaxy/, accessed on 11 January 2023). Functional prediction of gut microbes was performed based on the PICRUSt [26].

### 2.10. Statistical Analysis

This experiment was performed using GraphPad Prism 9.0.0 (GraphPad, San Diego, CA, USA) statistical software for the statistical analysis of the data. The statistical methods were mean ± standard error of the mean or the median ± interquartile range. A parametric analysis of differences between groups was performed using a one-way analysis of variance (ANOVA) with Dunnett’s multiple comparison test. Correlation analysis and visualization between indexes were performed using ChiPlot (https://www.chiplot.online/, accessed on 12 January 2023).

## 3. Results

### 3.1. B. bifidum CCFM1163 Relieved CC Symptoms in Senna Extract-Treated Mice

**Figure 2:**
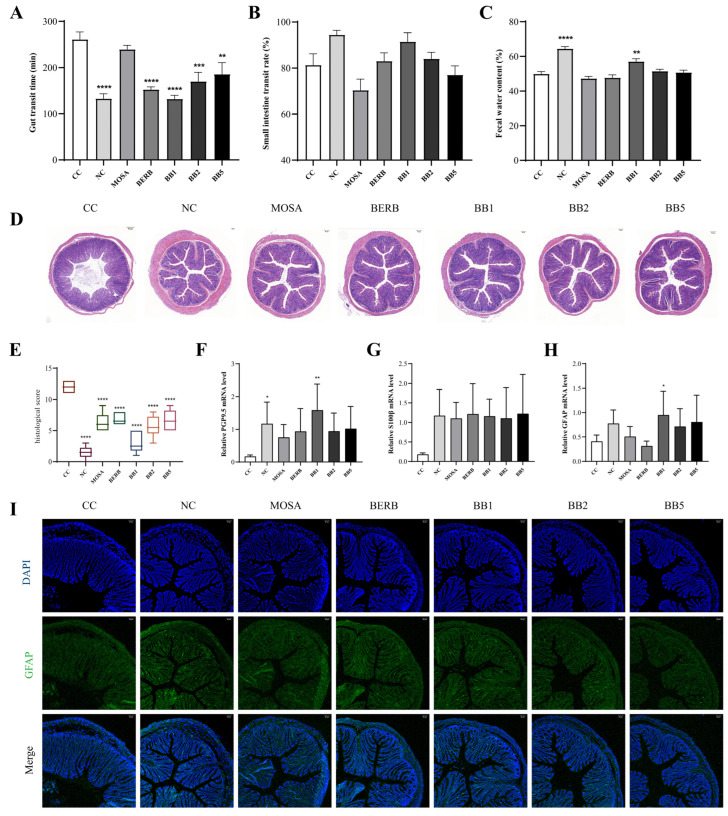
The effects of *B. bifidum* CCFM1163 on constipation and enteric nervous system-related indices. (**A**) Gut transit time. (**B**) Small intestine transit rate. (**C**) Fecal water content. (**D**) H&E stain. (**E**) Histopathological score. (**F**–**H**) Relative mRNA expression of PGP9.5, S100β, and GFAP in the colon. (**I**) The GFAP immunofluorescence map. Data are presented as the mean ± standard error of the mean or median ± interquartile range (n = 5–6). * *p* < 0.05; ** *p* < 0.01; *** *p* < 0.001; **** *p* < 0.0001 comparing with cathartic colon group.

Although senna extract-treated mice had lower fecal water contents and longer gut transit times than the NC group, the small intestine transit times were not markedly different among the groups (*p* > 0.05). These data demonstrated that the animal CC model can be successfully constructed using senna extract (Figure 2A–C). In addition, H&E staining (Figure 2D) and the histological injury score of the colon (Figure 2E) of the senna extract-treated group exhibited destroyed epithelium, decreased goblet cells, crypt loss, and the infiltration of inflammatory cells into the mucosal layer and even the submucosal layer. In addition, the results of the immunofluorescence analysis and mRNA expression levels of enteric nerve-specific markers suggested a notably decreased mRNA expression level of PGP9.5 in CC mice (*p* < 0.05, Figure 2F) and lower gene expression levels of GAFP and S100β in NC mice (*p* = 0.10, *p* = 0.07, Figure 2G–I). These results suggest that senna extract could damage the mechanical barrier and intestinal nerve in the mouse colon during the construction of an animal CC model.

BERB-, *B. bifidum* CCFM1163-, BB2-, and BB5-treated groups had significantly decreased gut transit times compared with the CC group. The *B. bifidum* CCFM1163- and BERB-treated groups displayed the best effect and restored the gut transit time to a normal pattern (Figure 2A). Additionally, although *B. bifidum* CCFM1163 increased the fecal water content, it was statistically different from the other groups (*p* < 0.05, Figure 2C). According to the evaluation index of positive results for the relief of constipation in the *Technical Specification for Evaluation of Health Food*, 2022 edition, *B. bifidum* CCFM1163 is known to relieve CC. We further performed a histopathological assessment of the distal colon tissue. Colonic tissue damage was observably repaired in CC mice treated with drugs and *B. bifidum* compared with those in the CC group, with a recovery of mucosa and crypt structure (Figure 2D–E). Injury to mouse colon tissue was quantified using the pathological score. As illustrated in Figure 2E, the colon histopathological scores of MOSA-, BERB-, *B. bifidum* CCFM1163-, BB2-, and BB5-treated groups were 52.8%, 58.3%, 26.7%, 47.2%, and 55.6% of the CC group, respectively. *B. bifidum* CCFM1163 intervention reversed the enteric nervous damage caused by senna extract compared with that in the CC group. This was mainly manifested by a remarkable increase in the mRNA expression levels of PGP9.5, GFAP (*p* < 0.05), and S100β (*p* = 0.13, Figure 2F–I) in colonic tissues. Considered together, these results suggest that *B. bifidum* CCFM1163 relieved CC and repaired the intestinal mechanical barrier and enteric nervous damage caused by senna extract.

### 3.2. Analysis of B. bifidum CCFM1163 Mechanism in CC Relief

#### 3.2.1. *B. bifidum* CCFM1163 Can Repair Intestinal Mechanical Barrier Damage by Promoting the Expression of TJ Proteins

**Figure 3:**
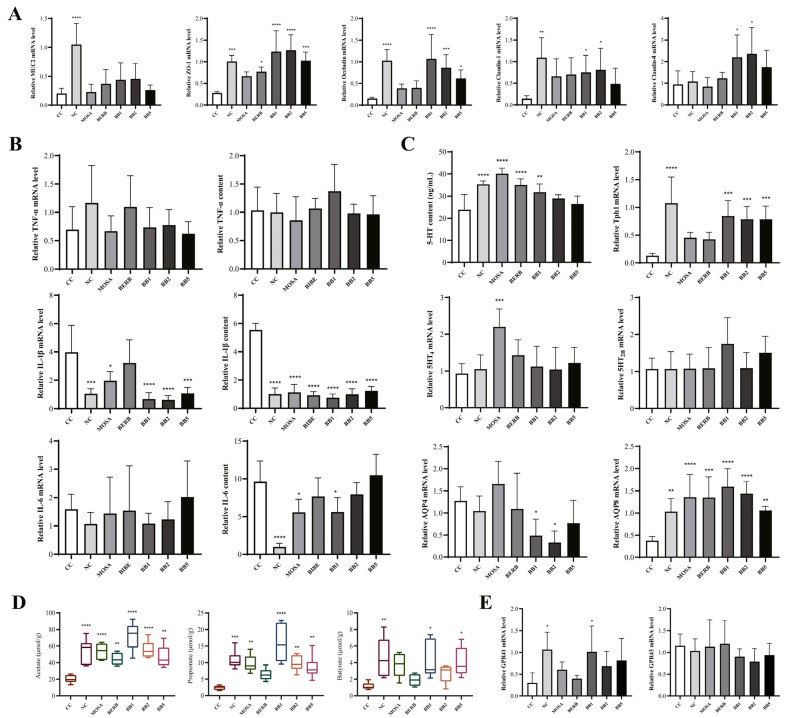
The effects of *B. bifidum* CCFM1163 on intestinal barrier-related indices. (**A**) Relative mRNA expression of MUC2, ZO-1, Occludin, Claudin-1, and Claudin-4 in the colon. (**B**) Relative mRNA expression and content of TNF-α, IL-1β, and IL-6 in the colon. (**C**) 5-HT content and relative mRNA expression of Tph1, 5-HT_4_, 5-HT_2B_, AQP4, and AQP8 in the colon. (**D**) SCFA concentrations in feces. (**E**) Relative mRNA expression of GPR41 and GPR43 in the colon. Data are presented as the mean ± standard error of the mean or median ± interquartile range (n = 5–6). * *p* < 0.05; ** *p* < 0.01; *** *p* < 0.001; **** *p* < 0.0001 comparing with cathartic colon group.

To investigate the effects of different bacterial strains on the intestinal mechanical barrier in mice, we determined the transcript levels of MUC2 and four TJ proteins in the colon. As illustrated in Figure 3A, the mRNA expression levels of MUC2, ZO-1, Occludin, and Claudin-1 were markedly decreased in the CC group (*p* < 0.05). These findings demonstrated that the application of senna extract to construct a constipation model was accompanied by damage to the intestinal mechanical barrier, mainly in the form of the thinning of the intestinal mucus layer and an increase in intestinal permeability. Compared with the CC group, almost all *B. bifidum* strain treatment groups notably increased the mRNA expression levels of ZO-1, Occludin, and Claudin-1 in the colon, as well as the mRNA expression level of MUC2 in the colon. These results demonstrated that *B. bifidum* has extremely beneficial effects in improving intestinal permeability and promoting colonic mucus secretion. These effects were not observed for the positive control drug. These results suggest that *B. bifidum* CCFM1163 alleviates CC while repairing the damage to the intestinal mechanical barrier caused by it.

#### 3.2.2. *B. bifidum* CCFM1163 Can Alleviate Intestinal Immune Barrier Inflammation by Reducing IL-6 and IL-1β Levels

The effects of senna extract treatment on the gene and protein expression levels of proinflammatory cytokines in mice are presented in Figure 3B. At the mRNA level, the relative expression levels of TNF-α, IL-1β, and IL-6 in the CC group were 0.60, 3.79, and 1.49 times higher than those in the NC group. At the protein level, the relative expression levels of pro-inflammatory cytokines in the CC group were 1.03, 5.54, and 9.63 times higher than those in the NC group. These data suggest that the application of senna extract also caused an inflammatory response in the host during the construction of a CC model. *B. bifidum* and drug intervention reduced the inflammation level in the organism to varying degrees; however, the effect of *B. bifidum* CCFM1163 in reducing the inflammation level was comparable with that of the positive drug Mosapride. These findings suggest that *B. bifidum* CCFM1163 reduces host intestinal inflammation and improves the immune barrier of the intestine while relieving CC.

#### 3.2.3. *B. bifidum* CCFM1163 Can Regulate Intestinal Chemical Barrier by Altering 5-HT and AQP Expression and Increasing SCFA Content in Feces

We determined the 5-HT content and mRNA expression of TPH1, 5-HT_2B_, 5-HT_4_, AQP4, and AQP8 in the colon. As presented in Figure 3C, the 5-HT content and gene expression levels of Tph1 and AQP8 were markedly reduced in CC mice (*p* < 0.05). These results suggest that senna extract inhibits the expression of Tph1, thereby reducing the neurotransmitter 5-HT content in the colon and that abnormal changes in 5-HT may be one of the neuropathological bases for slowed colonic transmission in CC mice. Moreover, after measuring the metabolites of the gut microbiota, the levels of AA, PA, and BA were found to be markedly downregulated in the intestine of mice treated with senna extract (Figure 3D). The specific receptor for SCFAs (GPR41) also exhibited a notably downward trend (Figure 3E). After different *B. bifidum* and drug interventions, the gene expression level of Tph1 in the colon markedly increased in all *B. bifidum*-treated groups (*p* < 0.05). There was also a corresponding statistically significant increase in 5-HT content in the colon, but only in the *B. bifidum* CCFM1163-treated group (*p* < 0.05). Furthermore, *B. bifidum* CCFM1163 markedly reduced the expression level of AQP4 and increased that of AQP8 in the intestine (*p* < 0.05). Meanwhile, the levels of SCFAs in the intestines of mice were upregulated. Only the levels of PA and BA in the BREB-treated group and BA in the MOSA- and BB2-treated groups were not statistically different from those in the CC group (*p* > 0.05). The above results suggest that *B. bifidum* CCFM1163 repaired damage to the intestinal chemical barrier while relieving CC.

#### 3.2.4. *B. bifidum* CCFM1163 Can Improve Gut Microbial Dysbiosis

**Figure 4:**
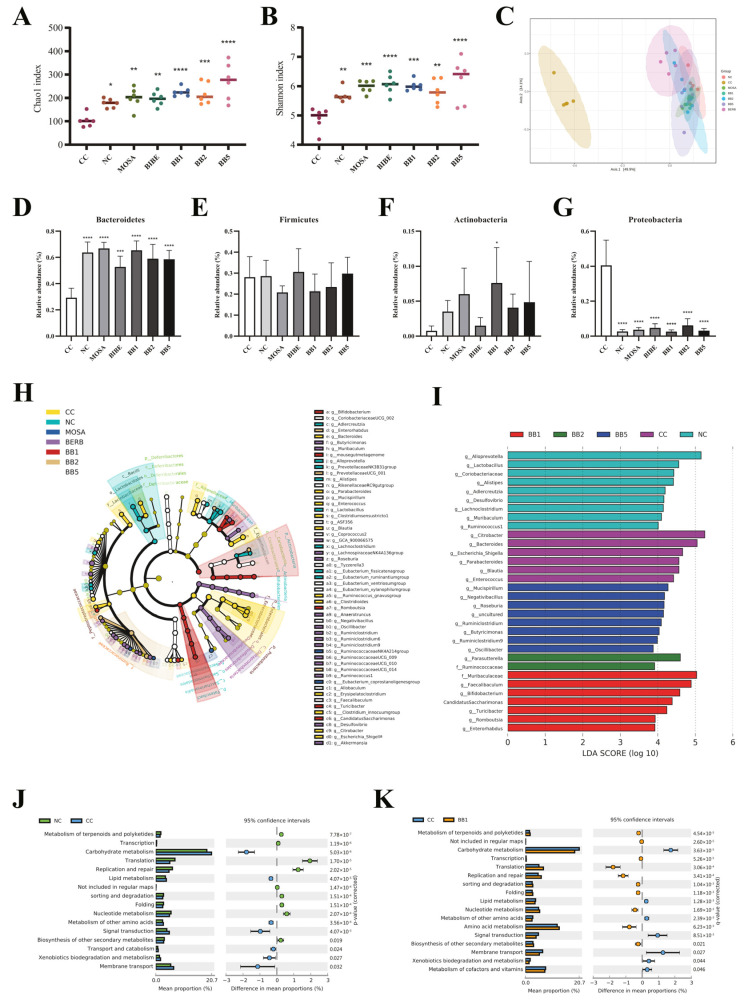
(**A**,**B**) The α-diversity indicted by Chao 1 and Shannon indices. (**C**) Beta diversity reflected by PCoA. (**D**–**G**) The relative abundance of Bacteroidetes, Firmicutes, Actinomycetes, and Proteobacteria. (**H**) LEfSe cladogram analysis at the genus level. (**I**) Distribution histogram based on LDA. LDA score > 3.0. (**J**) Differential microbial functions between cathartic colon and normal control groups. (**K**) Differential microbial functions between cathartic colon and *B. bifidum* CCFM1163-treated groups. Data are presented as the mean ± standard error of the mean (n = 5–6). * *p* < 0.05; ** *p* < 0.01; *** *p* < 0.001; **** *p* < 0.0001 comparing with cathartic colon group.

We observed a significant alteration in the structure of intestinal flora in the CC mice (Figure 4A–C). This is mainly reflected in the observably lower diversity of gut microbiota (e.g., considerably lower Chao1 and Shannon indices) and the CC group with its specific flora structure (β-diversity). At the phylum level, senna extract treatment statistically reduced the relative abundance of Bacteroidetes in mice intestine while increasing the relative abundance of Proteobacteria (*p* < 0.05). Both *B. bifidum* and drug interventions increased the relative abundance of Bacteroides and decreased the relative abundance of Proteobacteria. Notably, *B. bifidum* CCFM1163 remarkably increased the relative abundance of Actinomycetes (Figure 4D–G). At the genus level, the biomarkers for the CC group were *Citrobacter*, *Bacteroides*, *Escherichia-Shigella*, *Parabacteroides*, *Blautia*, and *Enterococcus*, whereas those for the NC group were *Alloprevotella*, *Lactobacillus*, *Coriobacteriaceae*, *Alistipes*, *Adlercreutzia*, and *Desulfovibrio*. *Muribaculaceae*, *Faecalibaculum*, *Bifidobacterium*, *Turicibacter*, *Romboutsia*, and *Enterorhabdus* were markedly enriched in the *B. bifidum* CCFM1163-treated group (Figure 4H–I). These findings revealed that senna extract treatment altered the structure of the gut microbiota of mice and that damage to the intestinal biological barrier was repaired to varying degrees after *B. bifidum* and drug interventions.

To further investigate the effect of *B. bifidum* CCFM1163 on the function of the fecal flora, the functional profiles of the microbiota were predicted based on PICRUSt analysis. Mice in the CC group exhibited different functional gene composition profiles compared with NC mice. Specifically, carbohydrate metabolism, xenobiotic biodegradation and metabolism, and lipid metabolism were upregulated, whereas nucleotide metabolism and biosynthesis of other secondary metabolites were downregulated in the CC group compared with the NC group (Figure 4J). Seventeen pathways were identified in the CC and *B. bifidum* CCFM1163-treated groups, among which, amino acid metabolism, nucleotide metabolism, and biosynthesis of other secondary metabolites were upregulated, whereas xenobiotics biodegradation and metabolism, and lipid and carbohydrate metabolism were downregulated in the *B. bifidum* CCFM1163-treated group (Figure 4K). Overall, *B. bifidum* CCFM1163 treatment reversed the alteration in fecal flora function in the senna extract-treated mice.

#### 3.2.5. Correlation Analysis Revealed That CC Relief Is Associated with Changes in Gut Microorganisms

**Figure 5:**
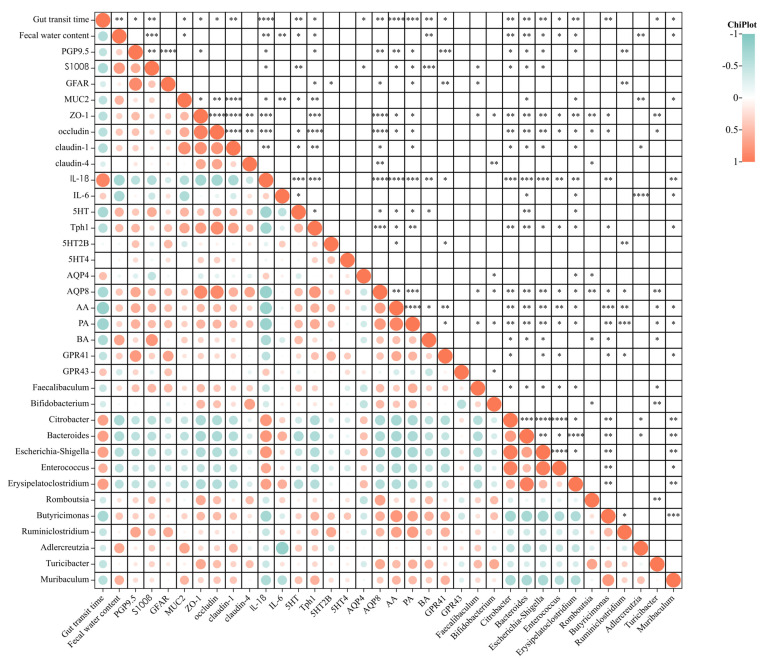
Correlation analysis between each index and gut microbiota. * *p* < 0.05; ** *p* < 0.01; *** *p* < 0.001; **** *p* < 0.0001.

We established correlations between the gut microbiota, CC apparent indices, and test indices based on the above results. The outcomes illustrated in Figure 5 indicate that IL-1β, AQP4, *Citrobacter, Bacteroides, Escherichia-Shigella, Enterococcus,* and *Erysipelatoclostridium* indices displayed a markedly positive correlation, and PGP9.5, S100β, MUC2, ZO-1, Occludin, Claudin-1, 5-HT, Tph1, AQP8, GPR41, AA, PA, BA, *Butyricimonas, Turicibacter,* and *Muribaculum* indices displayed a markedly negative correlation with intestinal transit time (*p* < 0.05). The S100β, MUC2, 5-HT, Tph1, BA, *Adlercreutzia,* and *Muribaculum* indices displayed a significantly positive correlation, and the IL-1β, IL-6, *Citrobacter, Bacteroides, Escherichia-Shigella, Enterococcus,* and *Erysipelatoclostridium* indices displayed a significantly negative correlation with the fecal water content rate (*p* < 0.05). In view of CC relief by *B. bifidum* CCFM1163, the correlation between each index after *B. bifidum* CCFM1163 treatment was analyzed. As previously described, *B. bifidum* CCFM1163 treatment notably increased the relative abundance of *Bifidobacterium*, *Faecalibaculum, Romboutsia,* and *Turicibacter* in the intestine (*p* < 0.05). ZO-1, Claudin-4, AQP8, and PA were significantly and positively correlated, whereas AQP4 and GPR43 were significantly and negatively correlated with *Bifidobacterium* (Figure 5). Similarly, S100β, GFAP, ZO-1, AQP8, and PA were positively correlated with *Faecalibaculum*. ZO-1, Occludin, AQP8, AA, PA, and BA were positively correlated with *Turicibacter.* ZO-1, Occludin, Claudin-4, AQP8, and BA were positively correlated, whereas AQP4 was negatively correlated with *Romboutsia* (*p* < 0.05).

In summary, these results suggest that a possible pathway, through which *B. bifidum* CCFM1163 alleviates CC, involves altering the gut microbiota, primarily by significantly increasing the relative abundance of *Bifidobacterium, Faecalibaculum, Romboutsia,* and *Turicibacter* in the feces. On the one hand, *B. bifidum* CCFM1163 increased SCFA content in feces, especially PA, thereby repairing the mechanical barrier of the intestine (increasing the expression of three TJ proteins, improving the absorption and secretion of water in the intestine, ultimately reducing the gut transit time, increasing fecal water content, and relieving CC constipation). On the other, it increased the relative abundance of *Faecalibaculum* in stools, increased the expression of enteric nervous marker proteins S100β and GFAP, repaired the ENS, and promoted intestinal motility, thus relieving constipation.

## 4. Discussion

In this study, a CC mouse model was used to systematically elucidate the pathogenesis of CC from both the intestinal barrier and ENS aspects for the first time, confirming the alleviating effects of *B. bifidum* CCFM1163 on CC. Based on this, we aimed to elucidate the mechanisms of CC alleviation by *B. bifidum* CCFM1163 and provide a theoretical basis for the development of probiotic formulations for CC.

Senna extract-treated mice had constipation symptoms of dry stool and impaired gut motility, confirming the successful establishment of the CC model in the treated animals. *B. bifidum* CCFM1163 treatment statistically reduced the total gut transit time, whereas no significant change was observed in the transit time of the small intestine. These results confirmed that *B. bifidum* CCFM1163 relieved CC symptoms owing to shortened colonic transit times. A previous study found that the long-term use of stimulant laxatives can damage the ENS, leading to impaired colonic motility, which is consistent with our results [27]. Moreover, *B. bifidum* CCFM1163 supplementation observably repaired the damaged tissue and reduced histological scores in the colon. This suggests that *B. bifidum* CCFM1163 effectively relieved CC symptoms. This study is the first to provide direct evidence of the role of *B. bifidum* CCFM1163 in alleviating CC.

A few studies have reported on the association between gut microbiota and ENS. Research on antibiotic-induced bacterial depletion mice has found that microbiota plays a vital role in the maintenance of ENS by regulating enteric neuronal survival and promoting neurogenesis [28]. Another study found that adding probiotics to the diet increases the expression of EGC marker proteins and neurotransmitters [17]. BERB has been reported to have enteric nerve repair effects [29] and therefore served as a positive drug control in this experiment. Our observations were consistent with those of two previous studies; *B. bifidum* CCFM1163 intervention notably increased the expression levels of enteric neurons and EGC marker proteins in the colon, and its effect was slightly better than that of BERB. In addition, *B. bifidum* CCFM1163 displayed anti-inflammatory effects in CC mice and played a role mainly in reducing IL-6 and IL-1β levels. IL-6 has been proven to exert either pro-inflammatory or anti-inflammatory properties, depending on its concentration and in combination with other inflammatory cytokines. Notably, the combination of high concentrations of IL-6 and IL-1β reduces neurogenesis [30]. These findings suggest a potential mechanism by which *B. bifidum* CCFM1163 affects the intestinal barrier and ENS.

Three mechanical barriers exist in the large intestine. Abundant goblet cells in the large intestine secrete mucin, organizing the mucous layer that covers the intestinal epithelium, which is the first mechanical barrier [31]. MUC2, the major mucin secreted in the intestine, plays an important barrier function, and mice lacking MUC2 develop spontaneous colitis [32]. Only a few bacteria have the enzymes required to metabolize mucin. Among *Bifidobacterium* species, only members of *B. bifidum* have been shown to degrade mucin. These enzymes can be used to produce SCFA during the fermentation process [33]. This may be the reason why *B. bifidum* CCFM1163 did not increase the expression level of MUC2 but increased the content of SCFA. The glycocalyx on intestinal epithelial cells provides the second mechanical barrier in the colon. The third mechanical barrier is the cell junction, which includes TJ proteins ZO-1, Occludin, and Claudin-1. *B. infantis* reduces colonic permeability and enhances the mechanical barrier by secreting an extracellular protein that promotes the expression of ZO-1 and Occludin [34]. We also confirmed that *B. bifidum* CCFM1163 exerts a protective effect on the intestinal barrier by increasing the expression of TJ proteins to repair intestinal mechanical barrier damage.

Enterogenous 5-HT is mainly generated by enterochromaffin cells under the action of TPH1 and promotes intestinal motility and secretion by binding to 5-HT-specific receptors [35]. However, different results have been reported regarding this issue. A few studies have reported that 5-HT is necessary for normal gastrointestinal motility [36,37], whereas others have indicated that 5-HT does not play a key role [38,39]. The cause of this phenomenon is the decreased level of 5-HT receptor 5-HT_2B_ in the colonic interstitial cells of Cajal, which impairs the responsiveness of diabetic mice to 5-HT. Impaired colonic motility in diabetic mice was improved by activating the 5-HT_2B_ receptor. In contrast, normal mice injected with 5-HT_2B_ receptor inhibitors exhibited a significant increase in colonic transit time [40]. To determine whether 5HT is involved in the regulation of intestinal motility in the CC model, we measured the 5HT content and the expression of related receptors. Interestingly, in this study, the 5-HT levels were notably reduced in the CC group; however, no significant differences were observed in the 5-HT_2B_ levels between the CC and NC groups. In addition, *B. bifidum* CCFM1163 promoted 5-HT secretion and increased 5-HT_2B_ expression. These results demonstrate that *B. bifidum* CCFM1163 may activate 5-HT_2B_ receptors by promoting 5-HT secretion, which, in turn, improves colonic motility in CC mice.

Generally, the increased expression of AQP4 and AQP8 is observed in mouse constipation models [41]. Nevertheless, we observed that the expression level of AQP8 was markedly reduced in the colon of CC mice compared with that in NC mice. Although the reason for these differences remains to be determined, they could be owing to AQP4 and AQP8 being expressed at different locations in the intestinal epithelium. AQP4 is immunolocalized to the basolateral membrane of colonic epithelial cells and can regulate water absorption in the intestine, whereas AQP8 is mainly located in the apical membrane and intracellular epithelial cells and regulates the transport of water [42,43]. H&E staining revealed that the colonic epithelial surface structure was severely damaged in the CC group, which might be responsible for the significant decrease in AQP8 expression in the apical membrane. However, the basal structure of the epithelium in the CC group was not damaged; therefore, the expression of AQP4 was not affected. Considered together, *B. bifidum* CCFM1163 may promote water secretion in the intestine by increasing the expression of AQP8, thus increasing fecal water content and alleviating CC.

As previously observed, the effect of probiotics on constipation relief is well established; however, controversy exists regarding the effect of these probiotics on SCFA production. Certain studies have reported a change in AA, PA, and BA [44,45], whereas others have not [46,47], which could be attributed to the characteristics of the strains. Our findings supported this hypothesis. Different strains of *B. bifidum* have different effects on SCFA. The effect of *B. bifidum* CCFM1163 on SCFA was greater than that of the other two strains, and it notably increased the contents of AA, PA, and BA in mouse feces. In addition, we discovered that the transcript level of the SCFA receptor GPR41 increased markedly after *B. bifidum* CCFM1163 intervention but not of GPR43. The selective signaling mechanisms of GPR41 and GPR43 differ markedly. GPR43 signaling involves L cell-derived peptide tyrosine, whereas GPR41 signaling involves submucosal neurons. The greatest FFA3 efficacy was observed in the terminal ileum and colon, in contrast with more uniform FFA2 signaling [48]. Correlation analysis also revealed that GPR41 levels were significantly positively correlated with ENS indicators. Considering that PA exhibits similar affinities for GPR43 and GPR41, we hypothesize that *B. bifidum* CCFM1163 activates the GPR41 receptor by increasing the level of PA in the colon, thereby regulating the ENS. We intend to explore this possibility in future research.

Recent evidence has revealed that the gastrointestinal microbiota plays a key role in gut motility. In clinical studies, fecal flora composition has been found to be associated with colonic transit time. The relative abundances of *Roseburia, Bacteroides, Lactococcus,* and *Actinobacteria* were related to faster gut transit time, whereas *Faecalibacterium* was directly associated with slower gut transit time [49]. Here, we found that the abundance of *Actinobacteria* and *Bacteroides* was reduced in model mice, and this trend was reversed by *B. bifidum* CCFM1163 treatment. We also observed an enrichment of certain pathobionts (*Escherichia-Shigella* and *Erysipelatoclostridium*) in the feces of model mice. It was documented that *Bifidobacterium* promotes intestinal motility by decreasing the abundance of *Alistipes, Odoribacter,* and *Clostridium* and increasing the abundance of *Lactobacillus* [4]. Moreover, *Bifidobacterium* can directly affect the biological barrier by reducing the abundance of potentially pathogenic bacteria, which may be related to the accompanying increase in SCFA levels [50]. In a separate study that used the same probiotic, this result was confirmed and was accompanied by an increased relative abundance of fecal bifidobacterial [51]. Interestingly, the fermentation end products of *Bifidobacterium* are AA and lactic acid, and lactic acid is readily converted to PA by other bacteria. Therefore, a notable increase in the content of *Bifidobacterium* in the *B. bifidum* CCFM1163-treated group may be one of the reasons for the higher levels of AA and PA in the feces.

Regardless of these interesting findings, a few questions remain to be addressed. First, we hypothesized that *B. bifidum* CCFM1163 acts by increasing the level of SCFAs in feces, but we did not verify this experimentally. Second, we only demonstrated that although *B. bifidum* CCFM1163 repairs damaged ENS, its possible pathway is unclear. Finally, further clinical trials are required to apply the results of animal experiments to clinical treatments.

## 5. Conclusions

In conclusion, this study determined that *B. bifidum* CCFM1163 effectively alleviated CC, and its main pathway involves changing the gut microbiota, significantly increasing the relative abundance of *Bifidobacterium, Faecalibaculum, Romboutsia,* and *Turicibacter* in the feces. This increased the SCFA content in feces, repaired intestinal mechanical barrier damage to promote intestinal transit, and regulated AQP8 expression in the intestine to increase fecal water content. Additionally, *B. bifidum* CCFM1163 increased the relative abundance of *Faecalibaculum* in the stool, increased the expression of S100β and GFAP, repaired the ENS, promoted intestinal motility, and relieved constipation.

## Data Availability

The datasets generated and analyzed during the current study are available from the corresponding author on reasonable request.

## Supplementary Materials

The following supporting information can be downloaded at https://www.mdpi.com/article/10.3390/nu15051146/s1. Table S1: Histopathological grading table. Table S2: Primer sequence.
