# Supplementary material for: Bifidobacterium bifidum CCFM1163 Alleviated Cathartic Colon by Regulating the Intestinal Barrier and Restoring Enteric Nerves
Source: Nutrients. 2023 Feb 24;15(5):1146. doi: 10.3390/nu15051146 (PMC10005791; doi:10.3390/nu15051146)
Supplement: Supplementary file 1 [file nutrients-15-01146-s001.zip › Supplementary Files.pdf]

Table S1. Histopathological grading table.

Inflammation	Extent	Crypt damage	Percent involvement (%)	Grade
None	None	None	None	0
Slight	Mucosa	1/3	1-25	1
Moderate	Mucosa and submucosa	2/3	26-50	2
Severe	Transmural	All	51-75	3
-	-	Entire crypt and epithelium lost	76-100	4

Table S2. Primer sequence.

Gene	Sequence (5' -> 3')	PrimerBank ID
PGP9.5	F-AGGGACAGGAAGTTAGCCCTA<br>R-AGCTTCTCCGTTTCAGACAGA	188219613c1
S100β	F-TGGTTGCCCTCATTGATGTCT<br>R-CCCATCCCCATCTTCGTCC	6677839a1
GFAP	F-CGGAGACGCATCACCTCTG<br>R-TGGAGGAGTCATTCGAGACAA	196115326c1
MUC2	F-AGGGCTCGGAACTCCAGAAA<br>R-CCAGGGAATCGGTAGACATCG	28865873a1
ZO-1	F-GCCGCTAAGAGCACAGCAA<br>R-GCCCTCCTTTTAACACATCAGA	254675278c1
Occludin	F-TTGAAAGTCCACCTCCTTACAGA<br>R-CCGGATAAAAAGAGTACGCTGG	6679162a1
Claudin-1	F-GGGGACAACATCGTGACCG<br>R-AGGAGTCGAAGACTTTGCACT	7710002a1
Claudin-4	F-ATGGCGTCTATGGGACTACAG<br>R-GAGCGCACAACTCAGGATG	160333265c1
TNF-α	F-CCCTCACACTCAGATCATCTTCT<br>R-GCTACGACGTGGGCTACAG	7305585a1
IL-1β	F-GAAATGCCACCTTTTGACAGTG<br>R-TGGATGCTCTCATCAGGACAG	118130747c1
IL-6	F-CTGCAAGAGACTTCCATCCAG<br>R-AGTGGTATAGACAGGTCTGTTGG	13624310c1
5-HT$_{2B}$	F-ACCTGATCCTGACTAACCGTT	118130975c1

	R-TGGGTATTATCACCGCGAGTAT	
5-HT4	F-AGTTCCAACGAGGGTTTCAGG<br>R-CAGCAGGTTGCCCAAGATG	6680325a1
AQP4	F-AGTCACCACGGTTCATGGAAA<br>R-CATGCTGGCTCCAGTATAATTGA	160415210c2
AQP8	F-TGTGTAGTATGGACCTACCTGAG<br>R-ACCGATAGACATCCGATGAAGAT	6680714a1
GPR41	F-CTTCTTTCTTGGCAATTACTGGC<br>R-CCGAAATGGTCAGGTTTAGCAA	142345193c1
GPR43	F-CTTGATCCTCACGGCCTACAT<br>R-CCAGGGTCAGATTAAGCAGGAG	22122727a1
Gapdh	F-AGGTCGGTGTGAACGGATTTG<br>R-GGGGTCGTTGATGGCAACA	126012538c1
